# Association of living alone with clinical outcomes in patients with heart failure: A systematic review and meta‐analysis

**DOI:** 10.1002/clc.24153

**Published:** 2023-09-22

**Authors:** Haibo Chen, Fuwei Liu, Jun Luo, Yating Tu, Shan Huang, Wengen Zhu

**Affiliations:** ^1^ Department of Psychiatry, Jiangxi Mental Hospital Affiliated Mental Hospital of Nanchang University Nanchang China; ^2^ Department of Cardiology The Affiliated Ganzhou Hospital of Nanchang University Ganzhou China; ^3^ Department of Psychiatry The Third People's Hospital of Ganzhou Ganzhou China; ^4^ Department of Cardiology the First Affiliated Hospital of Sun Yat‐Sen University Guangzhou China

**Keywords:** clinical outcomes, heart failure, living alone, meta‐analysis, systematic review

## Abstract

Living alone is an objective sign of social isolation. It is uncertain whether living alone worsens clinical outcomes in heart failure (HF) patients. We aimed to assess how living alone affected clinical outcomes in individuals with HF. We searched the electronic databases of PubMed, Embase, and Cochrane from 1990 to April 2022 for studies comparing living alone with HF. A random‐effects model with inverse variance was used to pool adjusted hazard ratios (HRs) and 95% confidence intervals (CIs). Seven studies were deemed to meet the standards. In patients with HF, compared with living with others, living alone was associated with an elevated risk of any hospitalization at the 30‐day (HR: 1.78, 95% CI: 1.09–2.89), 90‐day (HR: 1.24, 95% CI: 1.02–1.51), or ≥1‐year (HR: 1.14, 95% CI: 1.04–1.26) follow‐up periods. HF patients living alone also had a greater risk of any hospitalization or death at the 30‐day (HR: 1.56, 95% CI: 1.15–2.11), 90‐day (HR: 1.26, 95% CI: 1.05–1.50), and ≥1‐year (HR: 1.18, 95% CI: 1.09–1.28) follow‐up periods. However, patients living alone had no increased risk of all‐cause death at the 30‐day (HR: 1.0, 95% CI: 0.19–5.36), 90‐day (HR: 0.46, 95% CI: 0.03–7.42), or ≥ 1‐year (HR: 1.10, 95% CI: 0.73–1.67) follow‐up periods. In comparison to living with others, living alone was associated with an increased risk of any hospitalization but not all‐cause death in HF patients.

## Graphical abstract

This study sought to explore the association between living alone and adverse outcomes in patients with heart failure (HF). Pooled data showed that HF patients living alone had a higher risk of any hospitalization and any hospitalization or death during the 30‐day, 90‐day, and ≥1‐year follow‐up periods compared with living with others. Whereas in terms of all‐cause mortality, there was no difference in risk between the two groups.

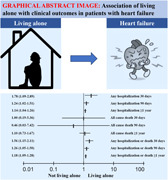

## 1 INTRODUCTION

Heart failure (HF) is currently a major global public health concern, which is associated with a greatly increased risk of adverse outcomes.^1^, ^2^ Despite significant progress in the standardized therapeutic management of HF, patients with HF continue to be at risk for cardiovascular events and hospitalization.^3^ Previous research has found that certain psychological factors such as sadness and anxiety are associated with increased risks of hospitalization or mortality in HF patients.^4^, ^5^ In recent years, some social risk factors (e.g., social isolation, inadequate social support, inadequate social networks, a lack of social relationships, and living alone) have been linked to a poor prognosis in HF patients with reduced ejection fraction (HFrEF) or preserved ejection fraction (HFpEF).^6^, ^7^, ^8^

Living alone is an objective indicator of social isolation.^9^ Previous research on the link between living alone and mortality has largely concentrated in the general population.^10^ However, few studies have assessed the implications of living alone in people with existing cardiovascular diseases. Giving persons good social support for HF could increase the quality of life, medication adherence, and proficiency.^11^ Living with a spouse or significant others is the most powerful type of social support. Several studies have examined the impact of social support on clinical outcomes in HF patients but have some limitations: (1) living alone is only one of many social lifestyle factors^12^, ^13^; (2) follow‐up is brief^12^, ^13^; and (3) HFrEF and HFpEF are combined in the analysis. Herein, we performed a systematic review and meta‐analysis aiming to assess whether living alone increases adverse outcomes in patients with HF.

## 2 METHODS

### 2.1 Search strategy

Our research complied with the statement on Preferred Reporting Items for Systematic Reviews and Meta‐Analyses.^14^ The PubMed, EMBASE, and Cochrane databases were systematically searched from 1990 to September 2022 for relevant studies that reported on the relationship between living alone and adverse outcomes in HF patients. The Boolean operators “and” were utilized in conjunction with the search topic terms: (1) heart failure or HF and (2) social isolation or living alone or living circumstances or living status. Supporting Information: Table 1 shows the exact search terms and strategy used for each database would improve the study's replicability. The language was not qualified by our search method. We checked the reference lists of the retrieved papers for further reported findings to confirm the thoroughness of the literature search.

### 2.2 Eligibility criteria

Studies were included if they met the following criteria: (1) study population: HF patients; (2) comparisons: living alone versus not living alone; (3) outcomes: death and/or hospitalization; (4) study designs: randomized controlled trials or observational studies; and (5) effect estimates: adjusted hazard ratios (HRs) and 95% confidence intervals (CIs). We excluded certain publications such as reviews, case series, case reports, letters, editorials, guidelines, and meeting abstracts. If the study participants came from the same database, the study with the longest duration or largest sample size was chosen.

### 2.3 Data extraction

All the retrieved records were screened by two independent authors. The included studies were all published in peer‐reviewed journals. According to the predefined criteria, we first read the titles and abstracts of the selected potentially eligible studies and then performed a more detailed review of the full texts. When facing discrepancies between authors, a third investigator was involved to establish a consensus for inclusion in the meta‐analysis. We extracted the following information for each study: first author and publication year, study location, design of the study, source of data, follow‐up period, baseline information, and adjusted HRs.

### 2.4 Quality assessment

We used the modified Newcastle‒Ottawa Scale (NOS) tool to assess the quality of observational cohort studies. This scoring scale consisted of three domains: cohort selection, cohort comparability, and outcome evaluation. The NOS ratings greater than 6 were deemed to indicate medium‐ to high‐quality.

### 2.5 Statistical analysis

We used Review Manager version 5.4 software to conduct a quantitative analysis (*Cochrane Collaboration 2014, Nordic Cochrane Centre Copenhagen, Denmark*; https://community.cochrane.org/). The Cochrane *Q* test and *I* ^2^ values were used to measure statistical heterogeneity among the included studies. The standard errors and natural logarithms of the included studies' HRs were calculated. A random‐effects model with an inverse variance method was used to pool all of the comparison results.^15^ We did not conduct sensitivity analyses to assess the robustness of the findings due to the limited data. When there were less than 10 included studies, the publication bias analysis should not be performed according to the Cochrane handbook for systematic reviews.^15^ As a result, we did not conduct a publishing bias analysis. In this study, the criterion for statistical significance was set at a two‐tailed *p*‐value < .05.

## 3 RESULTS

### 3.1 Study selection

**Figure 1:**
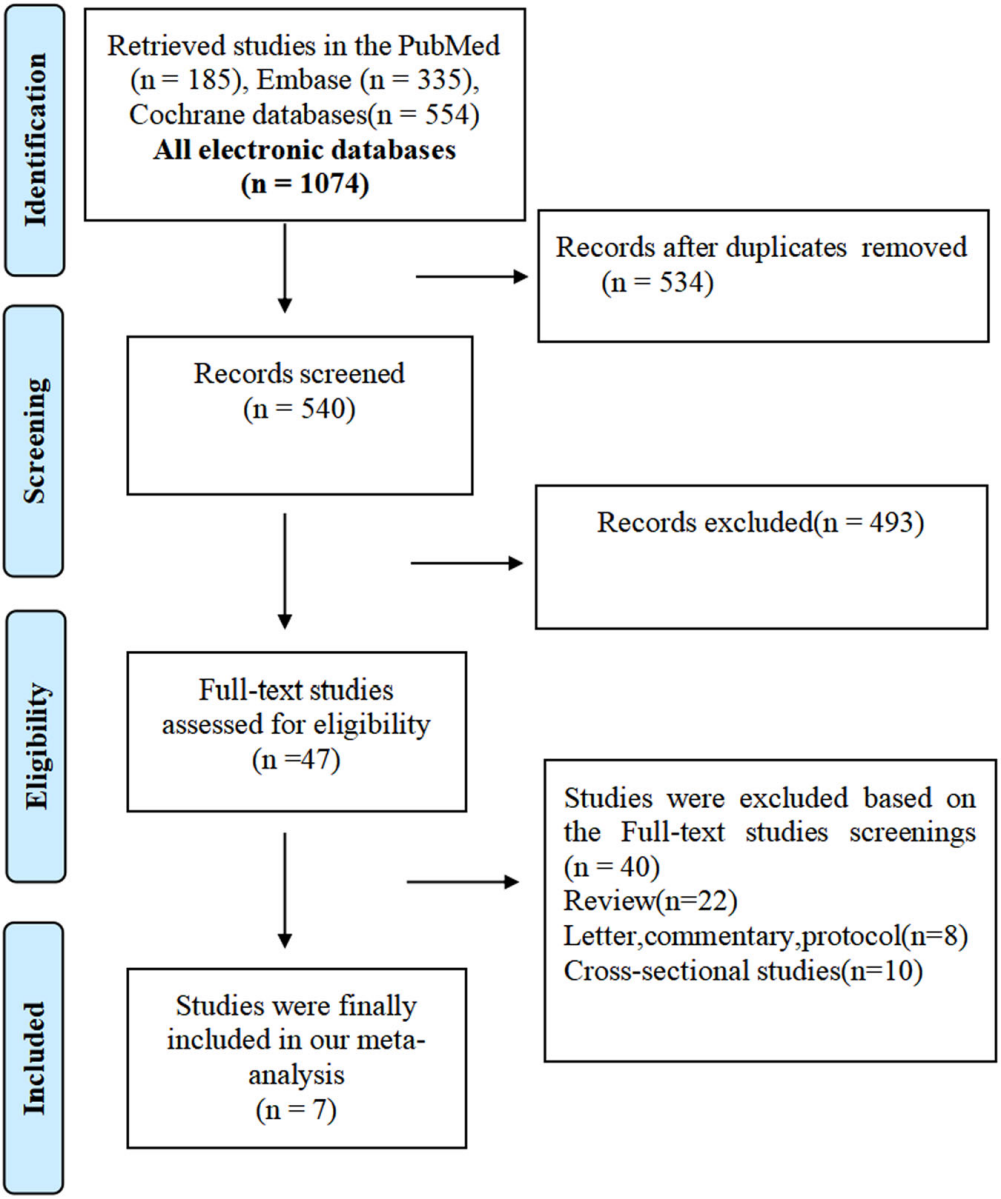
The process of the literature retrieval of this meta‐analysis.

As shown in Figure 1, a total of 1074 citations were obtained and extracted using our predefined search method. A total of 543 duplicate articles were eliminated. By analyzing the titles and abstracts of the literature using the prespecified inclusion and exclusion criteria, a total of 493 publications were discarded. The investigators meticulously studied the full texts of 47 studies, and 40 studies were further excluded. Of the remaining studies, seven articles^11^, ^12^, ^13^, ^16^, ^17^, ^18^, ^19^ were included in our meta‐analysis.

### 3.2 Study characteristics and quality assessment

Table 1 shows the baseline characteristics of the included studies. Four studies enrolled patients hospitalized for both HFpEF and HFrEF with an unknown left ventricular ejection fraction (LVEF) or a mean LVEF of <40%.^11^, ^12^, ^13^, ^17^ In contrast, Zhu et al. a post hoc analysis of the Treatment of Preserved Cardiac Function Heart Failure with an Aldosterone Antagonist trial and only included patients with HFpEF.^16^ Another study from Schockmel et al. also included only patients with HFpEF.^18^ A prospective, multicenter, community‐based cohort from Japan included only patients with acute HF.^19^ To assess the quality of cohort studies, we employed the NOS tool. Supporting Information: Table 2 summarizes the quality assessment outcomes for each included study. The included studies had moderate to high quality.

**Table 1:** Baseline patient characteristics of the included studies.

Author–publication year	Study location	Study design	Age, years		Female, %		LVEF, %		Sample size, N		Follow‐up time	Patients included	HF classification	Regression model	NOS
			Living alone	Not living alone	Living alone	Not living alone	Living alone	Not living alone	Living alone	Not living alone					
Sokoreli–2018	United Kingdom	Observational	NR	NR	NR	NR	NR	NR	NR	NR	1.0 y	Patients hospitalized for HF	Mixed	Cox regression	8
Huynh–2018	Australia	Observational	NR	NR	NR	NR	NR	NR	NR	NR	3.0 m	Patients hospitalized for HF during 2014–2017	Mixed	Logistic regression	7
Huynh–2015	Australia	Observational	NR	NR	NR	NR	NR	NR	NR	NR	1.0 m	Patients hospitalized for HF during 2009–2012	Mixed	Log‐binomial regression	7
Lu–2016	African America	Observational	65	69	41%	49%	31%	35%	242	369	1.0 y	Patients hospitalized for HF during 2011–2013	Mixed	Logistic regression	6
Zhu–2021	United States, Canada, Argentina, Brazil, Russia, and Georgia	Post hoc analysis of the TOPCAT trial	72	67	67%	43%	57%	56%	782	2321	3.3 y	Patients with symptomatic HFpEF	HFpEF	Cox regression	8
Schockmel–2014	France, Belgium and Luxembourg	Observational	NR	NR	NR	NR	NR	NR	NR	NR	1.5 y	Patients with HFpEF	HFpEF	Cox regression	6
Takabayashi–2020	Japan	Observational	75.8	74.4	44%	44%	53.3%	52.8%	124	457	3 y	Patients with AHF	AHF	Cox regression	7

Abbreviations: AHF, acute heart failure; EF, ejection fraction; HF, heart failure; HFpEF, heart failure patients with preserved ejection fraction; KICKOFF, Kitakawachi Clinical Background and Outcome of Heart Failure; LVEF, left ventricular ejection fraction; m, month; NOS, Newcastle Ottawa Scale; NR, not reported; ODIN, Observatoire de l'insuffisance cardiaque; TOPCAT, Treatment of Preserved Cardiac Function Heart Failure with an Aldosterone Antagonist; y, year.

### 3.3 Living alone and any hospitalization

**Figure 2:**
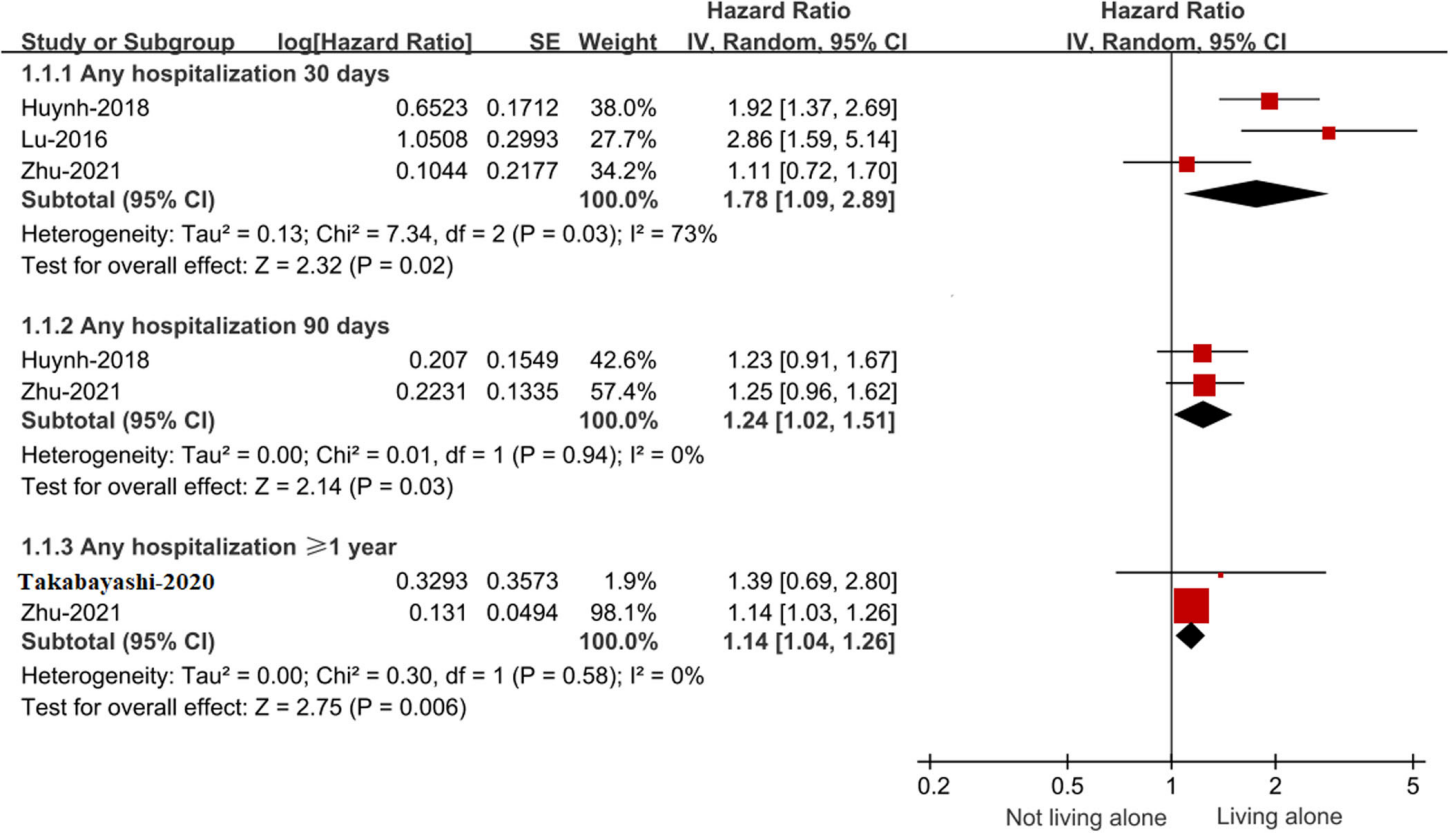
Forest plot for the effect of living alone on any hospitalization in patients with HF. CI, confidence interval; HF, heart failure; IV, inverse of the variance; SE, standard error.

According to the pooled effect estimates by the random‐effects model, living alone was related to an increased risk of any hospitalization in patients with HF during the 30‐day follow‐up period (HR: 1.78, 95% CI: 1.09–2.89), 90‐day follow‐up period (HR: 1.24, 95% CI: 1.02–1.51), and ≥1‐year (HR: 1.14, 95% CI: 1.04–1.26) follow‐up period (Figure 2).

### 3.4 Living alone and all‐cause death

**Figure 3:**
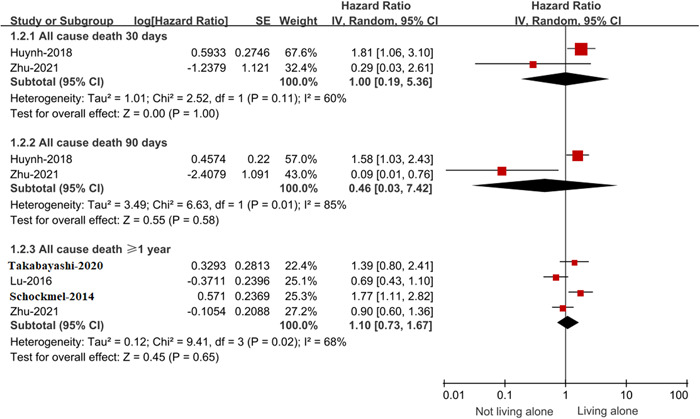
Forest plot for the effect of living alone on all‐cause death in patients with HF. CI, confidence interval; HF, heart failure; IV, inverse of the variance; SE, standard error.

Compared with HF patients living with others, patients living alone had no increased risk of all‐cause death at the 30‐day follow‐up period (HR: 1.0, 95% CI: 0.19–5.36), 90‐day follow‐up period (HR: 0.46, 95% CI: 0.03–7.42), and ≥ 1‐year (HR: 1.10, 95% CI: 0.73–1.67) follow‐up period (Figure 3).

### 3.5 Living alone and any hospitalization or death

Patients living alone had a higher risk of hospitalization or death compared with those living with others during the 30‐day follow‐up period (HR: 1.56, 95% CI: 1.15– 2.11), 90‐day follow‐up period (HR: 1.26, 95% CI: 1.05–1.50), and ≥ 1‐year (HR: 1.18, 95% CI: 1.09–1.28) follow‐up period (Supporting Information: Figure 1).

## 4 DISCUSSION

There were seven papers included in this analysis and the major indexes were hospitalization and/or death. The main findings of our current study were as follows: (1) living alone was associated with an elevated risk of any hospitalization at the 30‐day, 90‐day, and ≥1‐year follow‐up periods in HF patients; (2) living alone increased the risk of any hospitalization or death at the 30‐day, 90‐day, and ≥1‐year follow‐up periods in HF patients; and (3) living alone did not increase the risk of all‐cause death at the 30‐day, 90‐day, or ≥1‐year follow‐up periods in HF patients. Data should be interpreted cautiously due to the limited number of included studies.

Previous research has confirmed that a series of psychosocial factors such as cognitive impairment, depression, and anxiety are linked to an increased risk of hospitalization or death in patients with HF.^4^, ^5^ However, in the management of patients with HF, people always ignore the influence of social and psychological factors. Individualized treatment of HF patients should always be centered on their social background, and the overall risk factors should be controlled to improve their prognosis.^6^, ^7^ One of the most prevalent psychological variables is living alone. Living alone has been recognized as a major indicator of social isolation, defined as “the inadequate quality (subjective or perceived) and quantity (objective) of social relations with other people at the different levels where human interaction takes place.”^20^ Social isolation has been on the rise globally in recent years, which may raise the risk of hospitalization or mortality for patients with cardiovascular diseases, particularly those with HF. Living alone has resulted in less interaction with others, less social communication, and more social separation, all of which could result in objective social isolation. Evidence from previous studies has suggested that social isolation increases the risk of physical (e.g., cardiovascular disorders and diabetes mellitus) and mental health (e.g., depression and anxiety) disorders.^21^, ^22^ Living alone is associated with an increased risk of death in the general population.^9^, ^10^ Diabetes is more likely to be newly diagnosed and prevalent among those who live alone.^23^ Previous studies have revealed that living alone has an influence on the clinical prognosis of patients with established cardiovascular diseases. However, most of these investigations were conducted in individuals with stable or unstable coronary artery disease, and their results were disputed.^8^, ^24^, ^25^

More recently, seven studies assessed the relationship between living alone and the risk of hospitalization or death.^11^, ^12^, ^13^, ^16^, ^17^, ^18^, ^19^ Huynh et al. investigated the determinants of rehospitalization or mortality in patients hospitalized for HF in the 30 days after discharge (either HFpEF or HFrEF).^12^ Living alone raised the risk of hospitalization or death after adjusting for covariates. Furthermore, scientists analyzed another sample of HF patients and discovered that living alone was related to an increased risk of hospitalization and mortality at 30 or 90 days following the initial HF hospitalization.^13^ Sokol et al. investigated the predictive usefulness of psychosocial variables in patients with HF and discovered that people living alone had a 24% higher 1‐year hospitalization or death risk.^17^ Furthermore, Lu et al. discovered that patients with solitary HF had a higher 30‐day hospitalization rate, but there was no difference in the 1‐year risk of all‐cause death compared with those with nonsolitary HF.^11^ Zhu et al. included patients hospitalized for HFpEF and found a significantly increased risk of any hospitalization.^16^ Heidari et al.^6^ performed a qualitative review on the impact of living alone on hospital readmission in patients with HF. In contrast, we first performed a quantitative synthesis regarding living alone and clinical outcomes in HF patients.

Living alone was shown to be related to an increased risk of hospitalization or death in our current study. Nonetheless, we were unable to detect differences in the risk of all‐cause death between living alone and living with people. The possible reason given by Takabayashi et al.^19^ is that most of the patients included were elderly individuals, and they were older than the patients previously included by Japanese registries.^26^, ^27^ Elderly HF patients are more often exposed to the effects of various comorbidities and other risks inherent in old age. HF does not always immediately lead to death. HF patients have repeated exacerbations and remissions.^28^ Many comorbidities and other medical conditions could contribute to all‐cause death, especially in older HF patients, regardless of their living situation or support from others.

Social isolation, both objective (living alone) and subjective (loneliness), might damage cardiovascular health. Based on prior studies, living alone increased the risk of rehospitalization in HF patients, which may be due to multiple pathogenic mechanisms, including lifestyle changes (i.e., poor sleep quality and poor nutrition, low physical activity and cigarette smoking, poor treatment compliance), psychological disorders (i.e., depression and anxiety), or direct influences on biological factors (i.e., diabetes mellitus, hypertension, obesity, inflammation, and immunity).^22^ Living alone may result in hypothalamic‒pituitary‒adrenal axis activation, increased sympathetic activity, parasympathetic dysfunction, and proinflammatory immunological responses in terms of molecular pathways.^29^, ^30^ Some studies have suggested that oxidative stress caused by social isolation may have a deleterious influence on the development and progression of cardiovascular disease.^30^ Because the consequences of solitary life on cardiovascular disorders such as HF are complicated and multivariate, the previously indicated underlying processes are not causative. More evidence of this causal relationship is needed.

### 4.1 Limitations of the study

This study had several limitations that should be mentioned. First, given the included observational studies with limited evidence, further studies should take into consideration regarding the potential publication bias, the quality and heterogeneity of the included studies, and the absence of certain data that could have influenced the results. Second, the included studies were highly heterogeneous, with a small number of HF patients and no long‐term follow‐up results. We were unable to analyze the types of HF due to the small number of included studies. Third, it was unclear whether targeted interventions for people living alone would be beneficial based on the findings of this study. Intervention trials are required to determine whether interventions such as extra help at home, daycare, or remote monitoring are beneficial. Finally, we could not perform a quantitative synthesis regarding the outcomes of HF hospitalization and cardiovascular death in HF patients due to the limited data.

## 5 CONCLUSIONS

In comparison to living with others, living alone increased the risk of any hospitalization but not all‐cause death in HF patients. Due to the limited amount of available data, additional studies are required to confirm these findings.

## CONFLICT OF INTEREST STATEMENT

The authors declare no conflict of interest.

## Supporting information

Supporting information.

## Data Availability

The data could acquired from the corresponding authors with requests.
