# Supplementary material for: Association of living alone with clinical outcomes in patients with heart failure: A systematic review and meta‐analysis
Source: Clin Cardiol. 2023 Sep 22;47(1):e24153. doi: 10.1002/clc.24153 (PMC10765994; doi:10.1002/clc.24153)
Supplement: Supplementary file 1 — Supporting information. [file CLC-47-e24153-s001.docx]

**Data Supplement**

| **PubMed** | **Search terms** | **No.** |
| --- | --- | --- |
| #1 | Heart Failure | 139305 |
| #2 | Cardiac Failure OR Heart Decompensation OR Decompensation, Heart OR Heart Failure, Right-Sided OR Right-Sided Heart Failure OR Myocardial |  |
|  | Failure OR Congestive Heart Failure OR Heart Failure, Congestive OR Heart Failure, Left-Sided OR Left-Sided Heart Failure OR HF | 112969 |
| #3 | #1 or #2 | 204236 |
| #4 | social isolation OR living alone OR living status OR living arrangement | 16972 |
| #5 **Embase** | #3 and #4 | 185 |
| #1 | heart failure | 603977 |
| #2 | Cardiac Failure OR Heart Decompensation OR Decompensation, Heart OR  Heart Failure, Right-Sided OR Right-Sided Heart Failure OR Myocardial  Failure OR Congestive Heart Failure OR Heart Failure, Congestive OR Heart Failure, Left-Sided OR Left-Sided Heart Failure OR HF | 171712 |

**Supplementary Table 1. The search strategies of this meta-analysis**

| #3 #1 OR #2 | 659166 |
| --- | --- |
| #4 social isolation OR living alone OR living status OR living arrangement | 21507 |
| #5 #3 and #4  **Cochrane databases** | 335 |
| #1 heart failure | 41209 |
| #2 Cardiac Failure OR Heart Decompensation OR Decompensation, Heart OR Heart Failure, Right-Sided OR Right-Sided Heart Failure OR Myocardial  Failure OR Congestive Heart Failure OR Heart Failure, Congestive OR Heart Failure, Left-Sided OR Left-Sided Heart Failure OR HF | 25228 |
| #3 #1 OR #2 | 45387 |
| #4 social isolation OR living alone OR living status OR living arrangement | 13693 |
| #5 #3 and #4 | 554 |

**Supplementary Table 2. Quality assessment for the included studies**

Total

Selection (0-4 points) Comparability (0-2 points) Outcome (0-3 points)

points

| Included studies | Representativeness of Exposed Cohort | Selection of  Non-Exposed  Cohort | Ascertainment of Exposure | Demonstration That  Outcome of Interest  Was Not Present at  Start of Study | Adjust for the important  Risk factors | Adjust for other risk factors | Assessment of outcome | Followup length | Loss to follow-u p rate |  |
| --- | --- | --- | --- | --- | --- | --- | --- | --- | --- | --- |
| Sokoreli-2018 | * | * | * | * | * | * | * | * |  | 8 |
| Huynh-2018 | * | * | * | * | * | * | * |  |  | 7 |
| Huynh-2015 | * | * | * | * | * | * | * |  |  | 7 |
| Lu-2016 | * | * | * |  | * | * | * |  |  | 6 |
| Zhu-2021 | * | * | * | * | * | * | * | * |  | 8 |
| Matthieu-2014 | * | * | * |  | * | * | * |  |  | 6 |
| Kensuke-2020 | * | * | * | * | * | * | * |  |  | 7 |


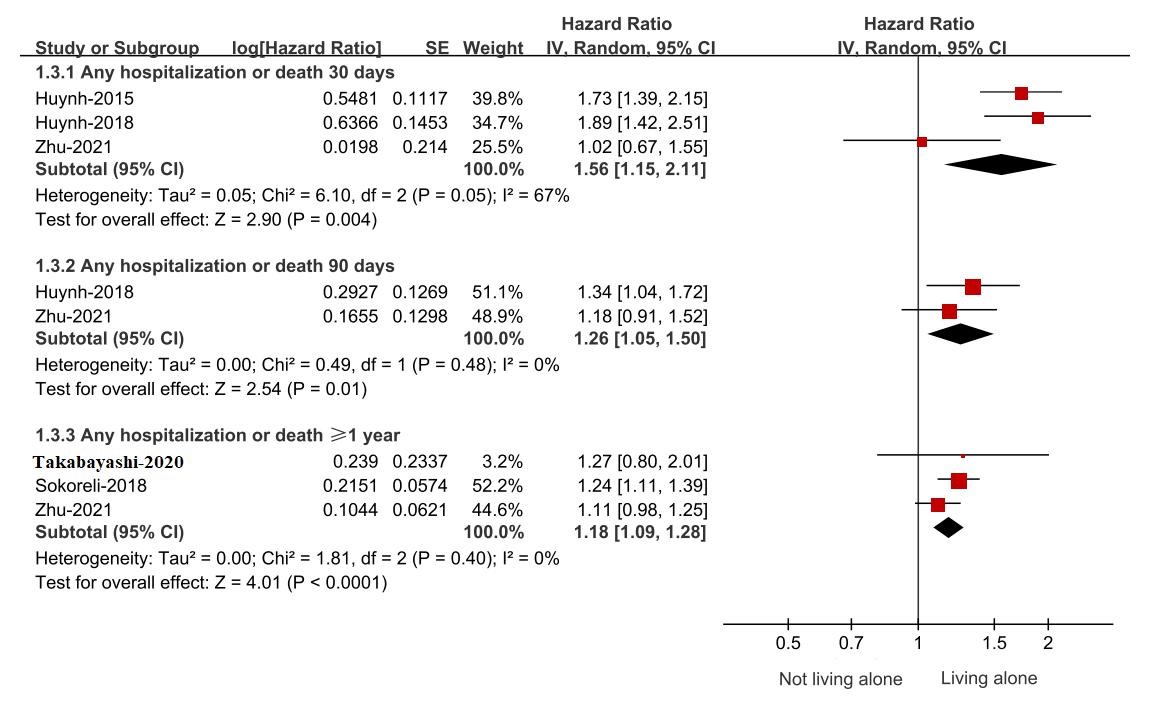


**Supplementary Figure 1. Forest plot for the effect of living alone on any hospitalization or death in patients with HF**

**Abbreviations**：**HF = heart failure; CI=confidence interval; SE=standard error; IV=inverse of the variance**
